# Cornel Iridoid Glycoside Attenuates Tau Hyperphosphorylation by Inhibition of PP2A Demethylation

**DOI:** 10.1155/2013/108486

**Published:** 2013-12-19

**Authors:** Cui-cui Yang, Xue-xian Kuai, Ya-li Li, Li Zhang, Jian-chun Yu, Lin Li, Lan Zhang

**Affiliations:** ^1^Department of Pharmacology, Xuanwu Hospital of Capital Medical University, Key Laboratory for Neurodegenerative Diseases of Ministry of Education, 45 Changchun Street, Beijing 100053, China; ^2^Tianjin University of Traditional Chinese Medicine, Tianjin 300193, China; ^3^First Teaching Hospital of Tianjin University of Traditional Chinese Medicine, Laboratory of Molecular Biology, Tianjin 300193, China

## Abstract

*Aim*. The aim of the present study was to investigate the effect of cornel iridoid glycoside (CIG) on tau hyperphosphorylation induced by wortmannin (WT) and GF-109203X (GFX) and the underlying mechanisms. *Methods*. Human neuroblastoma SK-N-SH cells were preincubated with CIG (50, 100, and 200 *µ*g/ml, resp.) for 24 h and then exposed to 10 *µ*M WT and 10 *µ*M GFX for 3 h after washing out CIG. Immunohistochemistry was used to observe the microtubular cytoskeleton of the cultured cells. Western blotting was used to measure the phosphorylation level of tau protein, glycogen synthase kinase 3**β** (GSK-3**β**), and protein phosphatase 2A (PP2A). The activity of PP2A was detected by a biochemical assay. *Results*. Preincubation of CIG significantly attenuated the WT/GFX-induced tau hyperphosphorylation at the sites of Thr205, Thr212, Ser214, Thr217, Ser396, and PHF-1 and improved the damage of morphology and microtubular cytoskeleton of the cells. CIG did not prevent the decrease in p-AKT-ser473 and p-GSK-3**β**-ser9 induced by WT/GFX. However, CIG significantly elevated the activity of PP2A by reducing the demethylation of PP2A catalytic subunit (PP2Ac) at Leu309 and the ratio of PME-1/LCMT in the WT/GFX-treated cells. The results suggest that CIG may be beneficial to the treatment of AD.

## 1. Introduction

Microtubule associated protein tau is mainly expressed in neurons and it is involved in modulating microtubule assembly and stabilizing the neuronal cytoskeleton [1]. The function of tau is accommodated by site-specific phosphorylation. Up to date, more than 40 phosphorylation sites of tau have been identified to be associated with Alzheimer's disease (AD) brain, such as Ser396, Ser404, and Thr212 [2, 3]. AD is the most common neurodegenerative disease in old people. Intracellular neurofibrillary tangles (NFTs) in brain is a major neuropathological hallmark of AD. Abnormal hyperphosphorylation of tau in the form of paired helical filaments (PHFs) is the main component of NFTs [4]. Hyperphosphorylated tau reduces the ability of tau in the assembly of microtubules and might lead to the destabilization of the neuronal cytoskeleton [5, 6].

The phosphorylation of tau is regulated by the activities of various kinases and phosphatases. Glycogen synthase kinase 3*β* (GSK-3*β*) is an important serine-threonine kinase that phosphorylates glycogen synthase in the glycogen synthesis pathway. Protein phosphatase 2A (PP2A) is a major phosphatase implicated in tau phosphorylation. Studies have shown that PP2A activity is decreased in AD brains [7]. GSK-3*β* and PP2A signaling pathways were reported to be interconnected. Activation of GSK-3*β* through simultaneous inhibition of phosphatidylinositol-3 kinase (PI3K) and protein kinase C (PKC) in rats or cells induces tau hyperphosphorylation [8–10]. Coadministration of wortmannin (WT, a specific PI3K inhibitor) and GF-109203X (GFX, a specific PKC inhibitor) induces tau hyperphosphorylation by activation of GSK-3*β*. Activation of GSK-3*β* can inhibit PP2A by increasing the inhibitory Leu309-demethylation involving upregulation of protein phosphatase methylesterase-1 (PME-1) and inhibition of leucine carboxyl methyltransferase (LCMT) [11]. PME-1 and LCMT catalyze the demethylation and methylation, respectively. However, there was no specific and effective drug to arrest tau hyperphosphorylation. Potentially novel strategies aimed at targeting tau pathology in neurodegenerative disease, suggesting that kinase inhibitors and phosphatase activators will be a potential therapy target [3].

*Cornus officinalis* Sieb. et Zucc is a member of the Cornaceae family. Use of this herb was first recorded in Shen Nong's Materia Medica about 2000 years ago in China. In traditional Chinese medicine, *Cornus officinalis* is used to tonify the liver and the kidney for the treatment of vertigo, aching back, spontaneous emission, and sweating. Clinically, *Cornus officinalis* is also used to treat AD combining with other herbs [12, 13]. Cornel iridoid glycoside (CIG) is a main component extracted from *Cornus officinalis*. The purity of CIG was 71.19% determined by RP-HPLC assay, in which morroniside accounted for 67% and loganin 33% [14]. Previous studies in our laboratory indicated that intragastrical administration of CIG dramatically improved neurological function and promoted neurogenesis and angiogenesis in the brain of rats with middle cerebral artery occlusion in both acute and chronic stages [14]. CIG also suppressed glial cell activation and inhibited neuronal apoptosis in the brain of cerebral ischemic rats [15]. Specifically, CIG effectively improved memory ability and promoted neuronal survival by increasing the expression of synaptophysin and neurotrophic factors in cholinergic deficit AD-like model rats [16]. All findings above indicated that CIG may play an important role in AD therapy.

In the present study, we treated the human neuroblastoma SK-N-SH cells with wortmannin and GF-109203X, the classical activator of GSK-3*β*, to induce AD-like tau hyperphosphorylation, and investigated the effects and mechanisms of CIG on tau phosphorylation.

## 2. Materials and Methods

### 2.1. Drug

Cornel iridoid glycoside (CIG) was extracted from *Cornus officinalis* as described previously and the purity of CIG was 71.19% determined by RP-HPLC assay, in which morroniside accounted for 67% and loganin 33% [14].

### 2.2. Antibodies and Chemicals

The primary antibodies used in this study are listed in Table 1. Wortmannin (WT) was purchased from Enzo Life Sciences (10 Executive Boulevard Farmingdale, NY, USA) and GF-109203X (GFX) was from Sigma Chemical Co. (St. Louis, MO, USA). RIPA lysis buffer was from Beyotime (Jiangsu, China); fetal bovine serum (FBS), Dulbecco's modified Eagle's medium (DMEM), and trypsin-ethylenediaminetetraacetic acid (EDTA) were from Gibco Invitrogen (Carlsbad, CA, USA); and bicinchoninic acid (BCA) protein quantitative analysis kit was from Applygen Technologies Inc. (Beijing, China).

### 2.3. Cell Culture and Treatment

Human neuroblastoma SK-N-SH cells were cultured in Dulbecco's modified Eagle's medium (DMEM) supplemented with 10% (v/v) fetal bovine serum (FBS) and were kept in a humidified atmosphere of 5% CO_2_ and 95% air at 37°C, and the culture medium was replaced every 3 days. SK-N-SH cells were treated for 24 h with different concentrations of CIG (50, 100, and 200 *μ*g/mL) and then exposed to 10 *μ*M wortmannin and GF-109203X in serum-free DMEM for 3 h after washing out CIG.

### 2.4. Immunocytochemistry for Microtubular Cytoskeleton

Cells growing on glass coverslips were fixed for 30 min at room temperature in 4% paraformaldehyde. Cell membranes were blocked in 3% bovine serum albumin for 1 h at room temperature and incubated with rabbit polyclonal *β*-tubulin (1 : 300 dilution) overnight at 4°C. Cells were washed and incubated with Oregon Green 488-conjugated goat anti-rabbit IgG secondary antibody (1 : 500) and visualized with a laser confocal microscope (Leica TCS SP5II, Wetzlar, Germany).

### 2.5. Western Blotting Assays for Tau Protein, GSK-3*β*, PP2Ac, and Related Factors

Total protein was extracted from cell lysates using RIPA buffer. Three volumes of cell homogenate were added to one volume of sample buffer then boiled for 5 min. The protein concentration was measured by RC-DC protein assay according to manufacturer's instructions (Bio-Rad, Hercules, CA, USA). The proteins were separated by 10% sodium dodecylsulfate-polyacrylamide gel electrophoresis (PAGE) and transferred to polyvinylidene fluoride membrane (Millipore, Bedford, MA, USA). The blots were then probed with primary antibodies and then incubated with the corresponding anti-mouse or anti-rabbit IgG horseradish peroxidase-conjugated secondary antibody and enhanced chemiluminescence kit (Pierce, Rockford, IL, USA). Densitometric quantification of the protein bands was analyzed by TINA software (Raytest Isotopenme Bgerate GmbH, Straubenhardt, Germany).

### 2.6. PP2A Activity Assay

PP2A activity was measured according to the PP2A assay protocol (V2460 kit, Promega, Madison, WI, USA). In brief, the extracts of cell samples were centrifuged to remove particulate matter, and then endogenous free phosphate was removed with gel columns. Enzyme samples (1–35 *μ*L) were incubated with a chemically synthesized phosphopeptide in the half area 96-well plate. After incubating at 37°C for 30 min, the reaction was stopped by adding 50 *μ*L of Molybdate Dye/Additive mixture and then the fluorescence intensity of the samples was measured under a 630 filter set.

### 2.7. Statistical Analysis

All results were expressed as mean ± SD. All data were analyzed using one-way analysis of variance (ANOVA) followed by Tukey's post-hoc test using SPSS16.0 software. A probability of *P* < 0.05 was considered statistically significant.

## 3. Results

### 3.1. CIG Prevents Morphological Damage Induced by WT/GFX in SK-N-SH Cells

**Figure 1:**
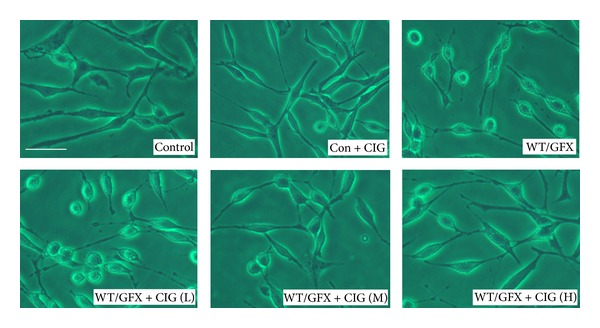
CIG prevents cell morphological damage induced by WT/GFX in SK-N-SH cells. The cell morphology was observed under contrast microscope. Control: normal human neuroblastoma SK-N-SH cells; Con + CIG: 100 *μ*g/mL CIG was incubated with normal SK-N-SH cells for 24 h; WT/GFX: SK-N-SH cells were exposed to 10 *μ*M wortmannin/GF-109203X (WT/GFX) for 3 h; WT/GFX + CIG (L, M, and H): SK-N-SH cells were preincubated with CIG (50, 100, and 200 *μ*g/mL) for 24 h and then exposed to 10 *μ*M WT/GFX for 3 h after washing out CIG. Bar = 50 *μ*m.

Figure 1 shows the morphology of SK-N-SH human neuroblastoma cells of the different groups under contrast microscope. Normal SK-N-SH cells spread well, and incubation of CIG did not influence the morphology of the normal cells. The cell bodies became round and the axons were shortened after the cells were exposed to 10 *μ*M wortmannin/GF-109203X (WT/GFX) for 3 h. Compared with WT/GFX-treated group, preincubation of CIG (100 and 200 *μ*g/mL) for 24 h with SK-N-SH cells prevented the damage induced by WT/GFX and improved the morphology of cells.

### 3.2. CIG Protects Microtubular Cytoskeleton against Injury Induced by WT/GFX

**Figure 2:**
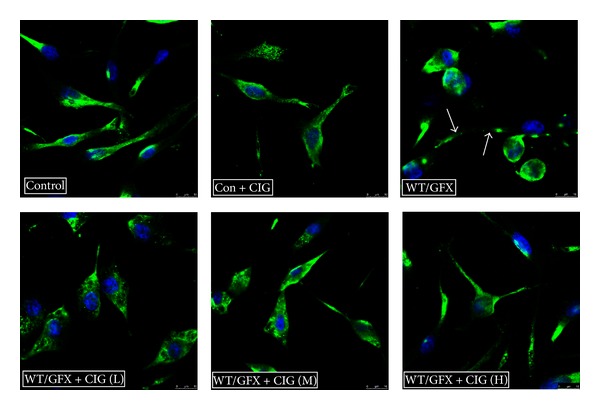
CIG protects microtubular cytoskeleton against injury induced by WT/GFX in SK-N-SH cells. Antibodies to *β*-tubulin and confocal fluorescence microscopy were used to visualize microtubular cytoskeleton. Control: normal human neuroblastoma SK-N-SH cells; Con + CIG: 100 *μ*g/mL CIG was incubated with normal SK-N-SH cells for 24 h; WT/GFX: SK-N-SH cells were exposed to 10 *μ*M WT/GFX for 3 h (the arrows point to the damaged microtubule cytoskeleton); WT/GFX + CIG (L, M, and H): SK-N-SH cells were preincubated with CIG (50, 100, and 200 *μ*g/mL) for 24 h and then exposed to 10 *μ*M WT/GFX for 3 h after washing out CIG. Bar = 50 *μ*m.

Tau hyperphosphorylation reduces its binding to tubulin and induces disruption of the microtubular cytoskeleton [17]. To observe the structures of microtubular cytoskeleton formed by tubulin, we performed confocal microscope analysis using antibodies to *β*-tubulin to visualize the structures. In the normal group, we found that *β*-tubulin (green) was distributed in cell bodies and processes homogeneously, and CIG did not affect the microtubular cytoskeleton of the normal cells. After being exposed to 10 *μ*M WT/GFX for 3 h, *β*-tubulin disappeared and the processes of cells were retracted, indicating that WT/GFX caused disintegration of the microtubular cytoskeleton. Preincubation of CIG (100 and 200 *μ*g/mL) with SK-N-SH cells for 24 h improved the structures of microtubular cytoskeleton compared with WT/GFX-induced model group (Figure 2).

### 3.3. CIG Inhibits Tau Hyperphosphorylation Induced by WT/GFX in SK-N-SH Cells

**Figure 3:**
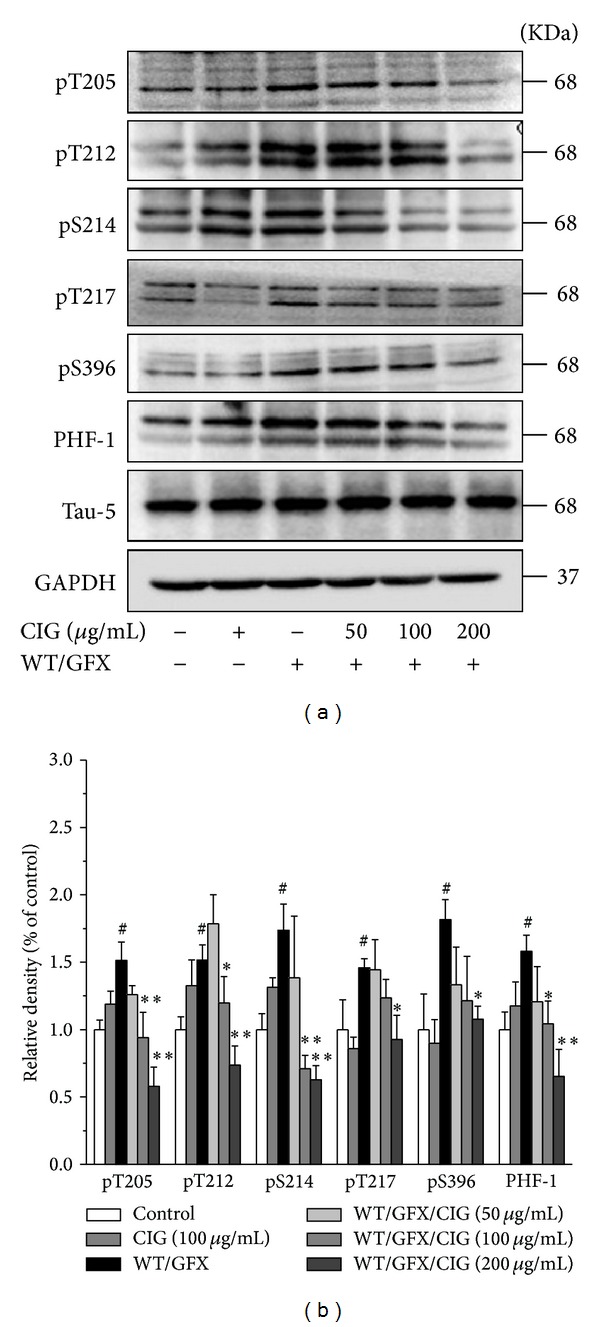
CIG reduces tau hyperphosphorylation induced by WT/GFX in SK-N-SH cells. SK-N-SH cells were pretreated with CIG (50, 100, and 200 *μ*g/mL) for 24 h and then exposed to 10 *μ*M WT/GFX for 3 h after washing out CIG. (a) The phosphorylation of different sites of tau protein was detected by Western blotting assay (including Thr205, Thr212, Ser214, Thr217, Ser396, and PHF-1). (b) Semiquantitative analysis of the levels of tau phosphorylation. GAPDH was used as an internal control. The level of tau phosphorylation of control group was set as 100%. Data were expressed as the mean ± SD of 3 experiments. ^#^ *P* < 0.05 versus control group; *P < 0.05, **P < 0.01 versus the WT/GFX model group.

The level of tau phosphorylation was measured by Western blotting analysis using antibodies that specifically recognize the different phosphorylation sites of tau protein. The results showed that the treatment of CIG for 24 h did not influence the normal cells. Compared with control group, 10 *μ*M WT/GFX treatment for 3 h obviously increased the levels of tau phosphorylation at the sites of Thr205, Thr212, Ser214, Thr217, and Ser396/404 (*P* < 0.05). Pretreatment of CIG (100 and 200 *μ*g/mL) for 24 h significantly attenuated the WT/GFX-induced tau hyperphosphorylation at Thr205, Thr212, Ser214, Thr217, and Ser396/404 (*P* < 0.05, *P* < 0.01) (Figure 3).

### 3.4. CIG Does Not Affect Akt/GSK-3*β* Signaling Pathway

**Figure 4:**
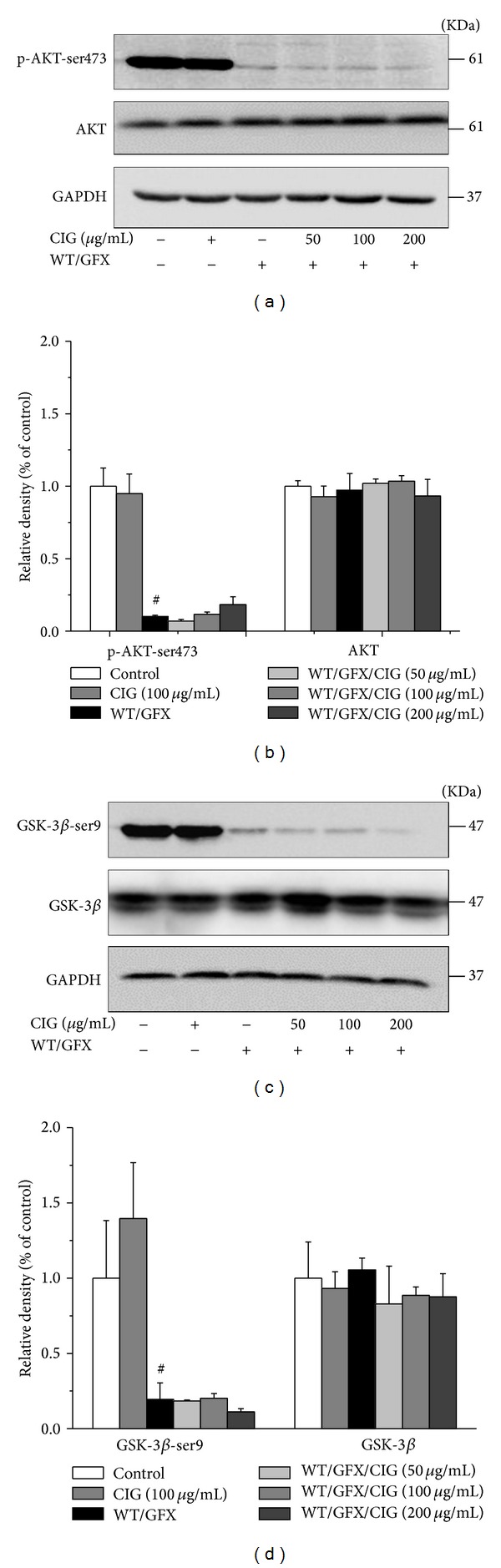
CIG does not alter the phosphorylation level of GSK-3*β* and AKT. SK-N-SH cells were exposed to 10 *μ*M WT/GFX for 3 h after the incubation with CIG (50, 100, and 200 *μ*g/mL) for 24 h. ((a), (b)) Western blotting analysis of the levels of phosphorylated Akt at Ser473 and total Akt; ((c), (d)) western blotting analysis of the levels of phosphorylated GSK-3*β* at Ser9 and GSK-3*β*. GAPDH was used as an internal control. The level of control group was set as 100%. Data were expressed as the mean ± SD of 3 experiments. ^#^ *P* < 0.05 versus control group.

GSK-3*β* is the pivotal kinase involving the formation of tau phosphorylation in AD brains. GSK-3*β* activity is regulated by Ser9 phosphorylation. Akt (protein kinase B) is upstream kinase in phosphorylating GSK-3*β* at Ser9 and inhibiting the activity of GSK-3*β* [18, 19]. To elucidate the mechanisms of effect of CIG on inhibition of tau phosphorylation, we measured Akt at Ser473 (the activated form) and GSK-3*β* at Ser9 (the inactivated form) in the cell extracts. The results displayed that the levels of phosphorylated Akt at Ser473 and phosphorylated GSK-3*β* at Ser9 were significantly decreased in the cells exposed to 10 *μ*M WT/GFX for 3 h (*P* < 0.05), suggesting that GSK-3*β* activity may be increased after PI3K/Akt inhibition. However, the pretreatment of CIG for 24 h did not alter the phosphorylation levels of both GSK-3*β*-ser9 and Akt-ser473 compared with WT/GFX model group. There was no obvious difference in the expression of GSK-3*β* and Akt among control, WT/GFX model, and CIG-treated groups (Figure 4). The results suggest that the inhibitory effect of CIG on tau phosphorylation may not be mediated by Akt/GSK-3*β* signaling pathway.

### 3.5. CIG Promotes the Activity of PP2A by Inhibiting PP2Ac Demethylation

**Figure 5:**
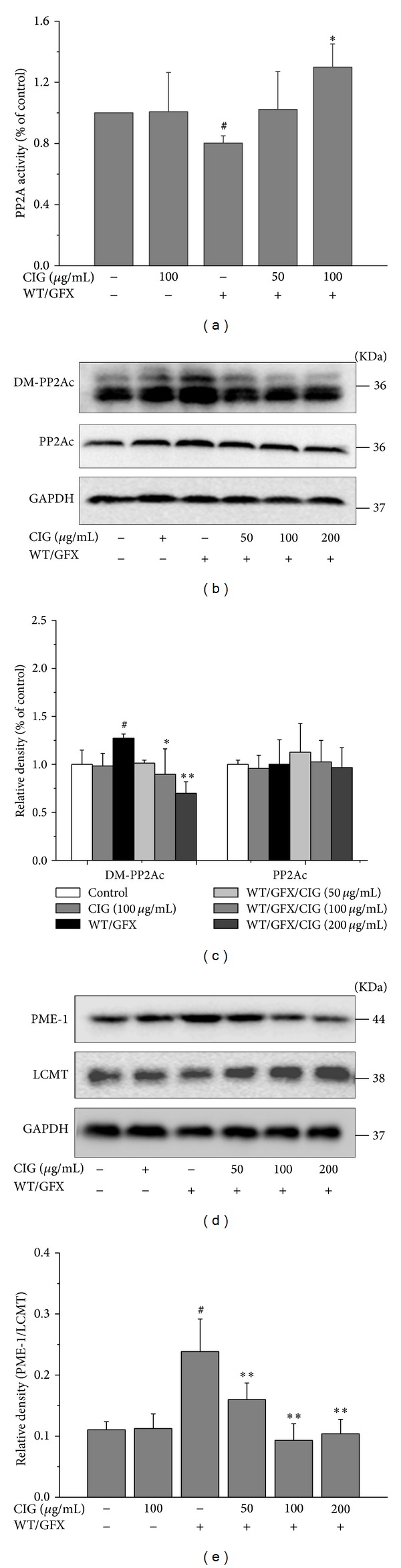
CIG promotes the activity of PP2A by inhibiting PP2Ac demethylation. SK-N-SH cells were preincubated with CIG (50, 100, and 200 *μ*g/mL) for 24 h and then exposed to 10 *μ*M WT/GFX for 3 h after washing out CIG. (a) The activity of PP2A measured by a biochemical assay, *n* = 6; ((b), (c)) the representative western blot image and the semiquantitative analysis of demethylation (DM) of PP2Ac at Leu307 and total PP2Ac; ((d), (e)) The representative western blot image of PME-1 and LCMT and the semiquantitative analysis of the ratio of PME-1/LCMT. GAPDH was used as an internal control. The level of control group was set as 100%. Data were expressed as the mean ± SD of 3 experiments. ^#^ *P* < 0.05 versus control group; *P < 0.05, **P < 0.01 versus the WT/GFX model group.

PP2A is an important phosphatase involved in dephosphorylation of tau. The activity of PP2A is also decreased via activating GSK-3*β*. Demethylation at Leu309 residue of PP2A catalytic subunit (PP2Ac) affects the activity of PP2A [20–22]. Thus, we detected the activity of PP2A and the expression of demethylated PP2Ac and total PP2Ac. The results showed that 10 *μ*M WT/GFX treatment for 3 h decreased PP2A activity in SK-N-SH cells compared with the control group (*P* < 0.05), and the pretreatment with 100 *μ*g/mL CIG significantly enhanced PP2A activity compared with the WT/GFX model group (*P* < 0.05) (Figure 5(a)). Although the expression of total PP2Ac appeared to be constantly expressed in all groups, the level of demethylated PP2Ac (inactive form) at Leu309 was obviously increased after the cells were exposed to WT/GFX (*P* < 0.05) but was significantly decreased in CIG-treated groups compared with the model group (*P* < 0.05, *P* < 0.01) (Figures 5(b) and 5(c)). It is known that PP2Ac demethylation is regulated by LCMT (a specific leucine carboxyl methyltransferase catalyzing methylation of PP2A) and PME-1 (a specific methylesterase catalyzing demethylation of PP2A) [21]. We found that WT/GFX increased the ratio of PME-1/LCMT (*P* < 0.05), which may enhance the demethylation level of PP2Ac. Pretreatment with CIG (50, 100, and 200 *μ*g/mL) significantly decreased the ratio of PME-1/LCMT compared with WT/GFX model group (*P* < 0.01) (Figures 5(d) and 5(e)). The incubation of CIG did not affect the above indicators in the normal cells.

## 4. Discussion

Tau hyperphosphorylation has been reported to play a pivotal role in AD pathology [23]. In the present study, we provided the evidence for the first time that CIG, an important ingredient of *Cornus officinalis*, inhibited hyperphosphorylation of tau via inhibiting PP2Ac demethylation.

The cytoskeleton is a cellular structure that provides neuronal morphology and whose essential components are the microtubules. The cytoskeleton is important in the formation of axon and dendrites, which are involved both in transport and neurotransmission [24]. Tubulin is the major building block of microtubules dynamic cytoskeletal structures involved in crucial cellular functions. The assembly and stability of microtubules are promoted by microtubule associated proteins [25]. Tau protein, a key microtubule associated protein (MAP), is a major protein that participates in the association-dissociation of the microtubules. Hyperphosphorylated tau tends to dissociate itself from microtubules and induces disruption of the microtubular cytoskeleton [26]. Tau is hyperphosphorylated at more than 40 sites in the AD brains [27–29]. In this study, we found that CIG protected microtubular cytoskeleton of cultured cells from disassembling induced by wortmannin and GF-109203X. Our results demonstrated that CIG attenuated the WT/GFX-induced tau hyperphosphorylation at Thr205, Thr212, Ser214, Thr217, Ser396, and PHF-1 (Ser396/404) sites in SK-N-SH cells. The mechanism of CIG maintaining the stability of microtubule cytoskeleton and thus improving the morphology of the neurons might be related to its dephosphorylation of tau. Overactivation of GSK-3*β* and downregulation of PP2A have been proposed to be involved in the abnormal tau phosphorylation in AD. The inactive form of GSK-3*β* which is phosphorylated at Ser9 is increased in AD brains [30]. Wortmannin (a specific inhibitor of phosphoinositol-3 kinase) and GF-109203X (a specific inhibitor of protein kinase C) activate GSK-3*β* activity by phosphorylating GSK-3*β* at Ser9 site. Phosphoinositol-3 kinase (PI3K) activates Akt, which inhibits GSK-3*β* by phosphorylating its Ser9 residue [9]. GSK-3*β* phosphorylates tau at many sites, with Thr205, Thr212, Ser214, Thr217, and Ser396/404 being the favorable sites in cells [31]. In the present study, we found that CIG reduced WT/GFX-induced tau hyperphosphorylation in SK-N-SH cells. However, CIG did not inhibit GSK-3*β* activity by increasing the phosphorylation at Ser9 and did not alter the phosphorylation level of AKT compared with WT/GFX model group.

PP2A is the major protein phosphatase in the brain that removes phosphate residues from tau, thereby stopping the ability of tau to inhibit microtubule assembly and to self-assemble into paired helical filaments and neurofibrillary tangles [32]. PP2A has been reported to dephosphorylate tau at several phosphorylation sites, and it might be a promising target to recover hyperphosphorylated tau in the AD brain to the normal tau. Among these sites, Thr205, Thr212, Ser214, Thr217, and Ser396/404 are also the favorable sites of PP2A [31, 32]. In the present study, we found that CIG decreased tau phosphorylation at these PP2A favorable sites. Some studies have suggested that GSK-3*β* and PP2A signaling pathways may be interconnected [31, 33]. GSK-3*β* inhibits the activity of PP2A via upregulating the demethylation of PP2A catalytic subunit (PP2Ac) at Leu309. Activation of GSK-3*β* by wortmannin increases the level of demethylation of PP2Ac at Leu309 [11]. In this study on mechanisms of CIG, we used the biochemical assay to explore PP2A activity. We found that the activity of PP2A was inhibited by WT/GFX indirectly, and CIG treatment enhanced PP2A activity compared with the model group. As expected, the demethylation of PP2Ac at Leu309 was increased by WT/GFX, and CIG treatment decreased the demethylation level of PP2Ac at Leu309. We propose that the enhancement effect of CIG on PP2A activity may be through its ability to reduce the demethylation of PP2Ac at Leu309.

Leucine carboxyl methyltransferase (LCMT) and protein phosphatase methylesterase-1 (PME-1) can catalyze the methylation and demethylation of PP2A, respectively [21]. Upregulation of GSK-3*β* induces the demethylation of PP2A at Leu309 by increasing the protein level of PME-1 and decreasing the protein level of LCMT [11, 34]. In our present study, the ratio of PME-1/LCMT was increased in SK-N-SH cells treated with WT/GFX, and CIG decreased the ratio of PME-1/LCMT compared with the model group. We speculate that the mechanism of CIG's decreasing demethylation of PP2Ac at Leu309 might be through regulating the expression of PME-1 and LCMT.

In conclusion, our findings provide the evidence in the first time that cornel iridoid glycoside (CIG) attenuates tau hyperphosphorylation by increasing the activity of PP2A. The mechanism of CIG may be related to decreasing the ratio of PME-1/LCMT, thus reducing the demethylation of PP2Ac at Leu309. The results suggest that CIG may be beneficial to the treatment of AD.

## Figures and Tables

**Table 1:** Primary antibodies employed in this study.

Antibody	Type	Specificity	Phosphorylation sites	Source
pT205	Poly-	P-tau	Thr181	Invitrogen
pT212	Poly-	P-tau	Thr212	Invitrogen
pS214	Poly-	P-tau	Ser214	Invitrogen
pT217	Poly-	P-tau	Thr217	Invitrogen
pS396	Poly-	P-tau	Ser396	Invitrogen
PHF-1	Mono-	P-tau	Ser396/404	Abcam
Tau-5	Mono-	Total tau		Calbiochem
Anti-p-GSK-3*β*	Poly-	P-GSK-3*β*	Ser9	Cell signaling
Anti-GSK-3*β*	Poly-	GSK-3*β*		Santa Cruz
Anti-p-AKT	Poly-	P-AKT	Ser473	Cell signaling
Anti-AKT	Poly-	AKT		Cell signaling
Demethylated PP2Ac	Mono-	Demethylated PP2Ac		Millipore
PME-1	Mono-			Santa Cruz
LCMT1	Mono-			Abcam
PP2A	Poly-	PP2A		Santa Cruz
*β*-Tubulin	Poly-	*β*-Tubulin		Sigma
GAPDH	Mono-	GAPDH		ZSGB-BIO

## References

[1] Chang E, Congdon EE, Honson NS, Duff KE, Kuret J (2009). Structure-activity relationship of cyanine tau aggregation inhibitors. *Journal of Medicinal Chemistry*.

[2] Wang J-Z, Grundke-Iqbal I, Iqbal K (2007). Kinases and phosphatases and tau sites involved in Alzheimer neurofibrillary degeneration. *European Journal of Neuroscience*.

[3] Hanger DP, Anderton BH, Noble W (2009). Tau phosphorylation: the therapeutic challenge for neurodegenerative disease. *Trends in Molecular Medicine*.

[4] Li B, Chohan MO, Grundke-Iqbal I, Iqbal K (2007). Disruption of microtubule network by Alzheimer abnormally hyperphosphorylated tau. *Acta Neuropathologica*.

[5] Noble W, Hanger DP, Miller CC, Lovestone S (2013). The importance of tau phosphorylation for neurodegenerative diseases. *Frontiers in Neurology*.

[6] Rodriguez-Martin T, Cuchillo-Ibáñez I, Noble W, Nyenya F, Anderton BH, Hanger DP (2013). Tau phosphorylation affects its axonal transport and degradation. *Neurobiology of Aging*.

[7] Boutajangout A, Sigurdsson EM, Krishnamurthy PK (2011). Tau as a therapeutic target for alzheimer’s disease. *Current Alzheimer Research*.

[8] Xu G-G, Deng Y-Q, Liu S-J, Li H-L, Wang J-Z (2005). Prolonged Alzheimer-like tau hyperphosphorylation induced by simultaneous inhibition of phosphoinositol-3 kinase and protein kinase C in N2a cells. *Acta Biochimica et Biophysica Sinica*.

[9] Liu SJ, Zhang AH, Li HL (2003). Overactivation of glycogen synthase kinase-3 by inhibition of phosphoinositol-3 kinase and protein kinase C leads to hyperphosphorylation of tau and impairment of spatial memory. *Journal of Neurochemistry*.

[10] Wang Y, Zhang J-X, Du X-X (2008). Temporal correlation of the memory deficit with Alzheimer-like lesions induced by activation of glycogen synthase kinase-3. *Journal of Neurochemistry*.

[11] Yao XQ, Li XC, Zhang XX (2012). Glycogen synthase kinase-3beta regulates leucine-309 demethylation of protein phosphatase-2A via PPMT1 and PME-1. *FEBS Letters*.

[12] Dou YC (2010). Clinical application analysis of cornus officimalis. *Journal of Shandong University of Traditional Chinese Medicine*.

[13] Liang JF, Qin C (2010). Advances in traditional Chinese medicine to treat Alzheimer's disease. *Shandong Journal of Traditional Chinese Medicine*.

[14] Yao R-Q, Zhang L, Wang W, Li L (2009). Cornel iridoid glycoside promotes neurogenesis and angiogenesis and improves neurological function after focal cerebral ischemia in rats. *Brain Research Bulletin*.

[15] Ya B-L, Li C-Y, Zhang L, Wang W, Li L (2010). Cornel iridoid glycoside inhibits inflammation and apoptosis in brains of rats with focal cerebral Ischemia. *Neurochemical Research*.

[16] Zhao L-H, Ding Y-X, Zhang L, Li L (2010). Cornel iridoid glycoside improves memory ability and promotes neuronal survival in fimbria-fornix transected rats. *European Journal of Pharmacology*.

[17] Reiniger L, Lukic A, Linehan J (2011). Tau, prions and A*β*: the triad of neurodegeneration. *Acta Neuropathologica*.

[18] Georgievska B, Sandin J, Doherty J (2013). AZD1080, a novel GSK3 inhibitor, rescues synaptic plasticity deficits in rodent brain and exhibits peripheral target engagement in humans. *Journal of Neurochemistry*.

[19] Takahashi M, Tomizawa K, Kato R (1994). Localization and developmental changes of *τ* protein kinase I/glycogen synthase kinase-3*β* in rat brain. *Journal of Neurochemistry*.

[20] Yoon SY, Choi HI, Choi JE, Sul CA, Choi JM, Kim DH (2007). Methotrexate decreases PP2A methylation and increases tau phosphorylation in neuron. *Biochemical and Biophysical Research Communications*.

[21] Bryant JC, Westphal RS, Wadzinski BE (1999). Methylated C-terminal leucine residue of PP2A catalytic subunit is important for binding of regulatory B*α* subunit. *Biochemical Journal*.

[22] Tolstykh T, Lee J, Vafai S, Stock JB (2000). Carboxyl methylation regulates phosphoprotein phosphatase 2A by controlling the association of regulatory B subunits. *The EMBO Journal*.

[23] Obulesu M, Venu R, Somashekhar R (2011). Tau mediated neurodegeneration: an insight into Alzheimer’s disease pathology. *Neurochemical Research*.

[24] Meraz-Ríos MA, Lira-De León KI, Campos-Peña V, de Anda-Hernández MA, Mena-López R (2010). Tau oligomers and aggregation in Alzheimer’s disease. *Journal of Neurochemistry*.

[25] Osiecka KM, Nieznanska H, Skowronek KJ, Jozwiak J, Nieznanski K (2011). Tau inhibits tubulin oligomerization induced by prion protein. *Biochimica et Biophysica Acta*.

[26] Iqbal K, Grundke-Iqbal I, Smith AJ, George L, Tung Y-C, Zaidi T (1989). Identification and localization of a *τ* peptide to paired helical filaments of Alzheimer disease. *Proceedings of the National Academy of Sciences of the United States of America*.

[27] Gong C-X, Liu F, Grundke-Iqbal I, Iqbal K (2005). Post-translational modifications of tau protein in Alzheimer’s disease. *Journal of Neural Transmission*.

[28] Liu F, Liang Z, Gong CX (2006). Hyperphosphorylation of tau and protein phosphatases in Alzheimer disease. *Panminerva Medica*.

[29] Hanger DP, Byers HL, Wray S (2007). Novel phosphorylation sites in Tau from Alzheimer brain support a role for casein kinase 1 in disease pathogenesis. *Journal of Biological Chemistry*.

[30] Lim Y-W, Yoon S-Y, Choi J-E (2010). Maintained activity of glycogen synthase kinase-3*β* despite of its phosphorylation at serine-9 in okadaic acid-induced neurodegenerative model. *Biochemical and Biophysical Research Communications*.

[31] Qian W, Shi J, Yin X (2010). PP2A regulates tau phosphorylation directly and also indirectly via activating GSK-3*β*. *Journal of Alzheimer’s Disease*.

[32] Liu F, Grundke-Iqbal I, Iqbal K, Gong C-X (2005). Contributions of protein phosphatases PP1, PP2A, PP2B and PP5 to the regulation of tau phosphorylation. *European Journal of Neuroscience*.

[33] Martin L, Page G, Terro F (2011). Tau phosphorylation and neuronal apoptosis induced by the blockade of PP2A preferentially involve GSK3*β*. *Neurochemistry International*.

[34] de Baere I, Derua R, Janssens V (1999). Purification of porcine brain protein phosphatase 2A leucine carboxyl methyltransferase and cloning of the human homologue. *Biochemistry*.

